# Molecular Imaging of Cancer with Nanoparticle-Based Theranostic Probes

**DOI:** 10.1155/2017/1026270

**Published:** 2017-06-19

**Authors:** Ying-Yu Ma, Ke-Tao Jin, Shi-Bing Wang, Hui-Ju Wang, Xiang-Min Tong, Dong-Sheng Huang, Xiao-Zhou Mou

**Affiliations:** ^1^Clinical Research Institute, Zhejiang Provincial People's Hospital, Hangzhou 310014, China; ^2^Key Laboratory of Cancer Molecular Diagnosis and Individualized Therapy of Zhejiang Province, Hangzhou 310014, China; ^3^Department of Gastrointestinal Surgery, Shaoxing People's Hospital, Shaoxing Hospital of Zhejiang University, Shaoxing 312000, China; ^4^School of Basic Medical Sciences, Hangzhou Medical College, Hangzhou 310053, China

## Abstract

Although advancements in medical technology supporting cancer diagnosis and treatment have improved survival, these technologies still have limitations. Recently, the application of noninvasive imaging for cancer diagnosis and therapy has become an indispensable component in clinical practice. However, current imaging contrasts and tracers, which are in widespread clinical use, have their intrinsic limitations and disadvantages. Nanotechnologies, which have improved in vivo detection and enhanced targeting efficiency for cancer, may overcome some of the limitations of cancer diagnosis and therapy. Theranostic nanoparticles have great potential as a therapeutic model, which possesses the ability of their nanoplatforms to load targeted molecule for both imaging and therapeutic functions. The resulting nanosystem will likely be critical with the growth of personalized medicine because of their diagnostic potential, effectiveness as a drug delivery vehicle, and ability to oversee patient response to therapy. In this review, we discuss the achievements of modern nanoparticles with the goal of accurate tumor imaging and effective treatment and discuss the future prospects.

## 1. Introduction

Although patient survival periods have improved, high five-year mortality rates are still associated with late-stage diagnosis such as metastasis [1]. Early diagnosis is closely related to survival rate for most cancer; for instance, 10-year survival for patients with early-stage of the breast, colorectal, and prostate cancer has a rate of about 80% [2]. Recently, the application of noninvasive imaging for cancer diagnosis and therapy is an essential component in the clinic. Widespread clinical imaging systems, including magnetic resonance imaging (MRI), computed tomography (CT), and ultrasonography (US) [3], provide only anatomic and physiologic information, but having their some intrinsic limitations such as imaging contrast and tracers makes them inconvenient due to their nonspecific distribution throughout the body, fast metabolism, and undesirable side effects [4–7]. Nanotechnology developments have made noninvasive diagnosis of molecular patterns with imaging systems feasible, by utilizing nanoparticles as contrast agents. Different nanoparticle types have been designed for the most popular modalities used for molecular imaging and it has been reviewed (Table 1) [8] and illustrated that the most appropriate modality has capability to identify precisely for a specific application.

Recently, multiple components loaded nanostructures, termed as theragnosis or theranostics [9], have been extensively tested as a strategy to achieve simultaneous cancer diagnosis and therapy. Interest in theranostic nanoparticles (NPs), acting as multifunctional nanosystems by integrating diagnostic and medicinal capabilities in a single nanoparticle, has grown significantly over the past decade [10–12]. Nanoparticles capable of targeting on a molecular level can be crucial in molecular process evaluation in a noninvasive manner, identifying precise cell types in vivo, accurately diagnosing molecular processes ex vivo, and targeting therapy [13–15]. One benefit of nanomedicine is that drug conjugated nanoparticles administered intravenously collect in the tumor via leaky tumor vasculature [16, 17] through a process called enhanced permeability and retention (EPR) effect. Although it varies among tumor types [18], they typically collect in sub-100 nm structures [19]. However, this method also results in a fraction of the nanoparticles entering healthy tissues, particularly the liver and spleen [20]. Thus, nanoparticle biocompatibility must also be considered.

Nanostructures are capable of delivery several probes for imaging, which may improve early-stage cancer identification by using multiple imaging modalities. The use of multicomponent nanoparticles for imaging with various modalities has the potential to conquer the limitations of single imaging modalities by improving resolution, tissue penetration depth, probe sensitivity, temporal resolution time, information providing, cost, and clinical relevance [21].

Functionalized nanoparticles have been revealed to act as carriers for drugs [22–24] and genes [25] and can be further covered with agents that target-specific molecular targets, like antibodies (Abs) [26–28] and aptamers (Aps) [29, 30], which could be used for diagnosis and targeted therapy. The overarching goal is to treat or reduce terminal illnesses, such as cancer, noninvasively, to reduce side effects [31]. Thus, to improve the targeted diagnostic and therapeutic efficacy of NPs, modifications of the nanoparticle surface with linkers and chelators may be vital.

The purpose of this paper is to explore the interface of cancer and nanoparticles and summarize the achievements of the current nanoparticles, especially in accurate cancer imaging and effective treatment. Furthermore, the prospects will also be discussed along with the clinical applications of nanoparticles in diagnosis and theranostics for cancer.

## 2. Computed Tomography

CT, measuring X-ray absorption using high-atomic number (*Z*) content material to improve the CT image sensitivity to targeted contrast agents, is the first method of choice for detection of cancer. The advantages of this modality include low cost, quick scan times, very high spatial resolution, and precise signal quantification. Low contrast agent accumulation leading to limited soft tissue discrimination has become one of the challenges in this field. Iodine, gold, bismuth sulfide, and composite ceramics with iron oxide and lanthanide materials are clinically used CT molecular imaging agents. The majority of CT molecular contrast agents have a maximum number of X-ray-absorbing atoms which are incorporated in a nanoparticle, at the desired emulsions ranging [32–34], liposomes [35], lipoproteins [36], and polymeric nanoparticles [37, 38].

Zheng et al. developed a novel lipid-based nanoliposomal imaging agent CF800 for NIR fluorescence imaging and CT imaging, which coencapsulated two commercially available agents approved by the Food and Drug Administration (FDA), indocyanine green (ICG), and iohexol [39]. Animal cancer models in mice (breast, ovarian cancers) and rabbits (lung, head, and neck) using CF800 demonstrated effective accumulation and visualization in these solid tumors [40, 41]. Patel et al. [42] revealed greater contrast imaging enhancement in lung tumors when CF800 was administered by CT image analysis, which exhibited the potentiality to demonstrate localization and visualization of CF800 in orthotopic lung cancer tumors. Nakagawa et al. [43] prepared PEG functionalized nanoparticles with 30 and 15 nm of gold (Au-PEG), conjugated with the anti-HER2 (human epidermal growth factor 2, a breast cancer biomarker) antibody via terminal PEG chains (Au-PEGHER2ab). The results showed that Au-PEG nanoparticles were capable of functioning as CT imaging contrast agent in breast cancer. However, the Au nanoparticles may present in the body for a long period of time, so it is necessary to examine the biodistribution of Au and analyze its safety.

Chen et al. [44] synthesized innovative iodinated gold nanoclusters (AuNCs@BSA-I) via bovine serum albumin (BSA) and chloramine-T, which represents remarkable biocompatibility, intense X-ray attenuation coefficient, and fluorescence/CT imaging ability. Then patient tissue derived xenograft (PDX) mouse model from human thyroid cancer was established for further study in translational application, and the results revealed that AuNCs@BSA-I exerts sensitive and accurate diagnosis characteristics. Moreover, AuNCs@BSA-I fluorescent/CT signals could distinguish minimal thyroid cancer, as small as 2 mm^3^, suggesting that AuNCs@BSA-I could potentially serve as a dual-mode fluorescent/CT imaging agent intended for early precise diagnosis of thyroid carcinoma, and had potential to be translated into clinical practice. Monodisperse spherical nanoparticles (GNCNs) are created in nonsevere conditions from gold nanoclusters (GNCs) (generated by Gadolinium (Gd^3+^) ions-induced assembly) under mild conditions which was reported with high X-ray attenuation for CT and possessed unique CT imaging ability in lung cancer cell A549 tumor-bearing mice [45]. Zhou et al. showed that folic acid- (FA-) conjugated silica capped gold nanoclusters were biocompatible and actively target the FA (+) MGC-803 cells and small (5 mm) tissues from gastric tumors in nude mice models in vivo [46]. This kind of nanoprobes showed high-quality CT imaging as well as red-emitting fluorescence imaging.

Additionally, some other new type CT contrast agents have been developed recently. WS_2_ nanosheets could be used as an X-ray computed tomography (CT) contrast agent for bioimaging of tumors [47]. Bovine serum albumin-coated WS_2_ nanosheets (BSA-WS_2_) were injected into nude mice bearing HeLa tumors, and strong signals from WS2 at the tumor site were clearly observed from the CT image [48]. Recently, oxygen-deficient tungsten oxide WO_2.9_ nanorods were reported to act as a promising theranostic agent for simultaneous CT imaging [49]. Rb_x_WO_3_ (rubidium tungsten bronze, Rb-TB) nanorods can be employed as a new dual-modal contrast agent for photoacoustic tomography (PAT) and CT imaging, which suggest possibility of the multifunctional Rb_x_WO_3_ nanorods for applications in cancer theranostics [50].

## 3. Magnetic Resonance Imaging

Compared to other imaging modalities, the advantages possessed by MRI are high soft tissue contrast and good spatial resolution. Additionally, MRI provides more viable and safe modality with vulnerable patients since MRI does not require radioisotopes or radiation. On the other hand, the insensitivity of magnetic resonance imaging (MRI) to contrast agents makes it an unideal modality for target-specific imaging. However, owing to the marked advances in MRI contrast agent design, molecular imaging using MR has become practical [51]. Molecular MR imaging contrast agents typically contain nanoparticulate probes with a high concentration of contrast-generating metals and hone in on a specific target with a ligand-bound contrast agent. Having its low sensitivity, MRI is limited by target-specific contrast agents in clinical application, but techniques that exploit amplification may solve this problem.

Superparamagnetic iron oxide nanoparticles (SPION) are the first objects which have been clinically approved for use as MRI contrast agents. SiO2 coated SPION core-shell nanoparticles labeled with near infrared fluorescence (NIRF) dye and anti-CD146 monoclonal antibody [52] could be used for NIRF imaging or MRI. It revealed that the gastric cancer xenograft model was identified 30 min after this nanoparticles administration.

Derivatized dextran coated magnetic nanoparticles [53] form a multipurpose platform for targeting ligand conjugation, as they support diagnostic imaging by MRI. Studies have showed that these nanomaterials are sufficiently not harmful and biodegradable [54, 55] and stay in the blood for an extended period of time. Experimentally dextran coated superparamagnetic iron oxide nanoparticles are a well-established platform for the generating multifunctional imaging agents like monocrystalline iron oxide nanoparticles (MION) [56, 57] and similar nanoparticles cross-linked to dextran (cross-linked iron oxide nanoparticles, or CLIO) to form substrates ready to be linked to targeting ligands. The MR imaging ability of these nanoparticles has led to the development of probes capable of imaging cellular and subcellular events with high resolution [58–61], allowing for early detection, prognosis, and cancer monitoring. For instance, MRI with lymphotropic superparamagnetic nanoparticles used with MION was sufficient to detect all patients with nodal metastasis although the sensitivity of node-by-node analysis was significantly higher than conventional MRI in prostate cancer [54], suggesting that using magnetic nanoparticles for high resolution MRI enables virtually undetectable prostate cancer lymph node metastases to be detected. 20 to 50 nm superparamagnetic MION are covered with varying thicknesses of dextran T10 to alter pharmacokinetic properties and macrophage recognition [54, 62]. Carboxymethyl dextran (polyglucose sorbitol carboxymethylether) nanoparticles with improved surface coatings, carrying higher iron payloads and capable of undergoing bolus injection, have also been developed.

Nanoparticle ferumoxytol, a third-generation magnetic nanoparticle, reduces immunologic sensitivity. This particle has an iron oxide core with a diameter of 6.8 ± 0.4 nm [63]. It was reported to modulate nodal signal intensity at the appropriate circulation interval, in order for malignant nodal activity to be detected by MRI [59], which may be used as a safer lymph node staging agent that is easier to deliver in prostate cancer. Magnetic nanoparticles (MNPs) introduced to the bloodstream by injection enhance MRI and provide a noninvasive and precise method of accurately evaluating vascular volume fraction (VVF) in various xenograft murine models, which is shown to be an alternative marker of microvessel density (MVD) and vessel development [64, 65]. Sonic hedgehog (Shh) expression promotes the formation and progression of pancreatic tumors and inhibits tumor cell death after treatment, demonstrating the vital function of Shh signaling in pancreatic tumor progression and survival [66]. Guimaraes et al. [58] imaged pancreatic ductal adenocarcinoma cell xenograft models with MRI enhanced with MNP following a treatment targeting the Hh pathway. The study revealed that MRI VVF and VVF quantity changes correlated with histopathologic indices of MVD viable gland index and proliferative index, which suggested that MRI VVF may serve as a surrogate marker of angiogenesis and an early predictive marker of therapeutic efficacy. Fluorescent magnetic nanoparticles conjugated by BRCAA1 monoclonal antibody were reported to target gastric cancer tissues in mice and could potentially be detected cancer by fluorescent imaging and MRI [67].

Superparamagnetic iron oxide nanoparticulate ferumoxtran-10 (Combidex) was highly effective at detecting metastatic lymph nodes in different cancer types. Report by Tatsumi et al. [68] also revealed that ferumoxtran-10-enhanced MRI effectively diagnoses gastric cancer lymph node metastases. However, despite this proven efficacy, ferumoxtran-10 has some logistical disadvantages, including the need for a slow infusion to minimize hypersensitivity-related side effects.

## 4. Ultrasonography Imaging

Recently, targeted US imaging (molecular US) with enhanced contrast has revealed itself to be a novel noninvasive molecular imaging strategy. US imaging has several advantages including transportability, cost-effectiveness, no ionizing irradiation involvement, better spatial and temporal resolution which allows for the images to be evaluated in real-time, global availability, and the capacity for molecular information extraction [69, 70]. The use of the latest US hardware with advanced contrast agent design is predicted to improve the sensitivity in assessing the targeted molecular expression, which would be used for clinical application in the near future [71].

A recent study found that drug delivery guided by imaging offered a noninvasive alternative to both surgical resection and systemic drug delivery for higher drug concentrations at tumor sites and side effects reduction [72]. Despite the known advantages of US, it could trigger drug release via inertial cavitation causing mechanical damage to the drug carriers [73]. US provided precise control over spatiotemporal drug release and drug transport into solid tumors, which were different from other stimuli including temperature, pH, and enzymatic degradation [74, 75].

The US intensity is easily adjustable according to the purpose, low intensities used for diagnosis (<720 mW/cm^2^), and high-intensity therapeutic irradiation (up to 10^5^ W/cm^2^) for tumor treatment [76]. Owing to the high loading capacity and easily adjustable composition and properties, polyelectrolyte multilayer microcapsules have emerged as promising US-sensitive drug delivery carries [77]. Chen et al. [78] demonstrated that hydrogen-bonded multilayers of tannic acid and poly(N-vinylpyrrolidone) (TA/PVPON) microcarriers had possessed a high US imaging contrast and could deliver encapsulated therapeutics under both low-intensity diagnostic (power intensities of 0.1 W/cm2) and high-intensity therapeutic (>10 W/cm2) US irradiation in tumor tissues. This provided insights for the design of theranostic microcarriers in imaging-guided US-triggered cancer therapy. Recent study showed that US contrast agents have been developed in quantification of angiogenesis and US imaging was used to assess tumor angiogenesis at a molecular level and in a noninvasive way [69]. Deshpande et al. [79] have evaluated tumor angiogenesis and associated markers by targeted microbubbles using US imaging. Microbubbles bound to antibodies against vascular endothelial growth factor receptor 2 were injected to murine tumor models and found that the US contrast agent bound to the antibodies showed significantly higher adherence to tumor blood cells [80]. Yang et al. [81] showed that interleukin-4 receptor-targeted liposomal doxorubicin promoted targeted drug delivery using US in brain tumor animal models.

An innovative ultrasound-mediated chemotherapy method was established by systemic injection of phase shift drug-loaded nanodroplets, which could vaporize into microbubbles under the action of US. Acoustic phase shift nanodroplets effectively accumulated in tumor tissue by indirect or direct targeting and then converted into microbubbles in situ by US [82]. Expansion of nanodroplets from acoustic droplet vaporization (ADV) induces mechanical tissue erosion and cell damage [83] and promoted vascular permeability and ultrasound ablation for tumor tissue [84, 85]. Ultrasound-responsive nanodroplets comprise a perfluorocarbon (PFC) core and a solid shell composed of lipids, polymer, and/or proteins. Various PFC nanodroplet formulations for drug and gene delivery have been generated by ultrasound controlled. Most of them comprised a block copolymer shell such as poly(ethylene oxide)-co-poly(L-lactide) (PEG-PLLA) or poly(ethylene oxide)-co-poly(caprolactone) (PEG-PCL) [86, 87], a lipid shell (DPPC, DSPE-PEG/cholesterol) [88], a protein shell (lung surfactant, albumin) [89–91], or a surfactant shell (perfluorooctanoic acid) [92].

Baghbani et al. [93] developed smart curcumin-loaded chitosan/perfluorohexane nanodroplets capable of several functions. These nanodroplets were developed for contrast-ultrasound imaging and evaluated its cytotoxicity in vitro on 4T1 human breast cancer cells. In effects of curcumin-loaded nanodroplets the cell growth was significantly decreased by ultrasound exposure, which suggested that curcumin-loaded chitosan/perfluorohexane nanodroplets might have great potential for imaged-guided cancer therapy.

Another contrast agent, fluorescent nanobubbles (NBs), was engineered for targeted US breast cancer imaging. NBs are made by capturing liquid tetradecafluorohexane (C6F14) inside a biodegradable photoluminescent polymer (BPLPs). This is done through an emulsion-evaporation process. The product is then linked with PNBL-NPY ligand in order to target Y1 receptors overexpressed in breast tumors [94]. This developed PNBL-NPY modified NBs exhibit excellent aqueous stability, photostability, low toxicity, and improved contrast ability for US imaging of Y1R-overexpressing breast cancer, which provides a novel nanoplatform that can be used to detect early-stage cancer and for treatment.

Ma et al. [95] constructed a double-targeted nanoparticle: monomethoxypoly(ethylene glycol)-poly(lactic-co-glycolic acid) (mPEG-PLGA) was modified by double-targeted antibody, anticarcinoembryonic antigen (CEA) and anticarbohydrate antigen 19-9 (CA19-9), and encapsulated with antitumor drug paclitaxel (PTX). The results showed that much more NPs may be facilitated to ingress the cells or tissues with US or US targeted microbubble destruction (UTMD) transient sonoporation in vitro, and US contrast-enhanced images revealed NPs with prolonged imaging time in nude mice of pancreatic cancer, which make it possible to further enhance antitumor effects by extending retention time in the tumor region. This novel double-targeted NPs capable of ultrasound contrast-enhanced imaging and antitumor therapy may be promising in clinic.

## 5. Positron Emission Tomography

Positron emission tomography (PET) is commonly used to diagnose abnormalities at the cellular/molecular level by providing quantitative imaging [96–98]. Highly specific radiopharmaceutical activity is utilized to obtain quality images for diagnosis [99, 100]. Although PET is suitable for monitoring biological processes with high sensitivity and specificity, high cost limited its clinical application. In the emerging era of increased personalization of oncology treatments, nanoparticles can provide an extremely useful tool for cancer treatment and subsequent follow-up monitoring.

**Figure 1:**
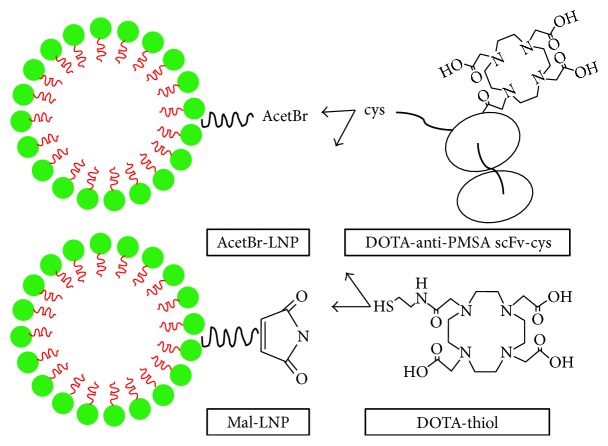
Schematic diagram of LNP constructs. DSPE-PEG-acetBr (acetBr-LNP) and DSPE-PEG-maleimide (mal-LNP) were conjugated to the DOTA-anti-PSMA scFv-cys or DOTA-monoacetamidoethanethiol (DOTA-thiol). LNP, lipid nanoparticles; DSPE, distearoyl phosphatidylethanolamine monomethoxy; PEG, polyethylene glycol; DOTA, 1,4,7,10-tetraazacyclododecane-1,4,7,10-tetraacetic acid; PSMA, prostate specific membrane antigen.

A single chain against prostate membrane antigen (PSMA) was conjugated to the copolymer, DSPE-PEG maleimide, that spontaneously assembled into a homogeneous multivalent lipid nanoparticle [101] (Figure 1) and then was expressed and evaluated by ^64^Cu PET imaging in a prostate cancer xenograft model, and the results revealed that the targeted anti-PSMA scFv-LNP showed enhanced tumor accumulation, which may provide evidence for targeted therapy of this system in drug delivery. In the area of cancer treatment, PET is primarily used to find localized radiolabeled nanoparticles for nanoparticle-mediated photothermal cancer therapy [30, 102]. Therefore, PET could potentially be used to assess patient response to treatment in order to improve patient outcome, reduce costs, and reduce time. [F-18] PET tracer fluoro-D-glucose (18F-FDG) is commonly used due to the high metabolism of tumor cells, and it has been used to diagnose tumors and evaluate treatment response [103, 104].

Jørgensen et al. [105] developed a single particle and PET-based platform in order to associate plasmonic nanoparticle heat with their ability to kill cancer cells. They investigated the effect of nanoparticle generated heat generation on human lung carcinoid tumor xenografts in mice with 2-deoxy-2-18F-FDG PET imaging. The research team found that PET imaging successfully tracked patient response to photothermal treatment in the early stages of the cancer. This interdisciplinary method provides a way to assess and compare emerging plasmonic nanoparticles for their potential as a cancer therapy.

Nanomaterials are commonly used to target angiogenic markers on tumor vasculature [106]. G-protein coupled transmembrane receptor follicle-stimulating hormone receptor (FSHR) is a common receptor concentrated in the vasculatures of primary tumors and metastatic sites [107–109]. PET imaging using FSHR targeting was first demonstrated using ^18^F-labeled FSH *β* 33–53 (a FSH fragment) in prostate tumors [110]. ^64^Cu-labeled monoclonal antibody (mAb) used to image FSHR in tumors via PET imaging further showed the value of FSHR as a cancer tissue marker [111]. FSH fragments-conjugated polymer [112] or dendrimer [113] based nanomaterials improve drug delivery to ovarian cancer cells by binding to FSHR-positive ovarian cancer cells. Utilizing monoclonal antibody against FSHR (FSHR-mAb) on polyethylene-glycol- (PEG-) functionalized graphene oxide (GO) nanosheets and ^64^Cu as a radiolabel to visualize GO conjugate distribution via PET imaging, Yang et al. [114] showed metastatic tumor targeting of GO conjugates in breast cancer and lung metastasis mouse models and high specificity for FSHR. Serial PET imaging also found that tumors take up ^64^Cu-NOTA-GO-FSHR-mAb and this marker stays stable over time and this FSHR-targeted, GO-based nanoplatform could be used for early metastasis detection and drug delivery.

## 6. Prospect and Conclusion

**Figure 2:**
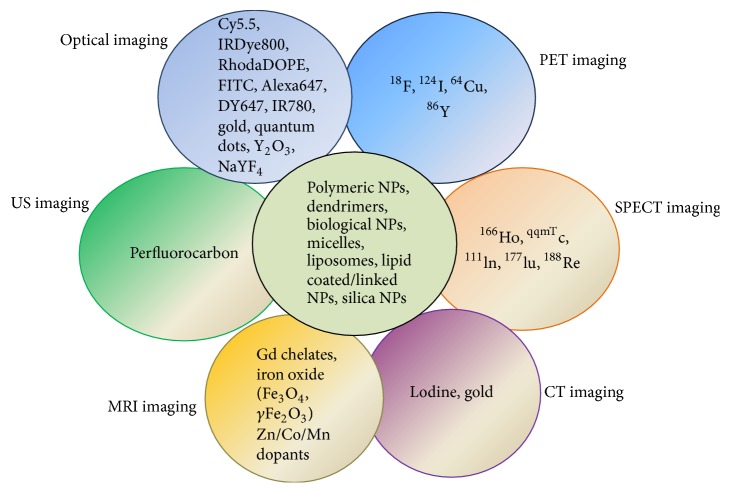
Incorporation of multicomponent imaging agents with various nanoparticles for multimodal imaging.

Nanoparticles applications of theranostics or multimodal imaging, which offers the possibilities to surpass these limitations of single imaging modalities, have been well-studied. To date, various combinations have been reported that cover dual-modal, trimodal, or other imaging modalities, such as MR-optical imaging [115], MRI-PET [116], optical imaging-CT [117], and MRI-CT [118] (Figure 2) [119].

A modern multifunctional drug carrier for image guided catheter-directed procedures is critically needed in order to improve therapeutic outcomes. Incorporation of imaging agents into the drug source itself (i.e., a radiopaque/magnetic microspheres) should offer several advantages over current embolization agents not visible with clinical imaging modalities [120]. Multimodal MRI/CT visible microspheres would be able to permit direct visualization of these drug carriers during the delivery of the antitumor drugs.

MRI/CT visible microspheres with gold nanorods and magnetic clusters were engineered, and the drug carriers would be best suited for administration by an intra-arterial catheter to liver tumors while allowing for imaging to verify tumor-targeted delivery [121]. MRI was used for identifying tumor regions and MRI/CT was used to confirm successful microspheres delivery to the targeted HCC following selective arterial infusion, which should allow timely prediction of therapeutic outcome and patient prognosis.

Photoacoustic imaging (PAI), which is an emerging, hybrid, and noninvasive biomedical modality, has been extensively explored for its applications in cancer imaging [122, 123], and exogenous contrast agent is preferably used to achieve high sensitivity PAI at the cellular level [124, 125]. Large amount of nanoparticles has been used for PAI, such as plasmonic gold nanoparticles (AuNPs) [125, 126] and plasmonic titanium nitride nanoparticles (TiN NPs) [127].

Following the disclosure of human genome, individualized medicine combining with targeted imaging and therapy toward neoplasm is in great demand. However, the combined treatment agent was not possible until the development of theranostic nanomedicine was fulfilled. The adenovirus (Ad), a vector commonly used for cancer gene therapy is limited in its therapeutic application by low coxsackievirus and adenovirus receptor (CAR) expression in tumors and its inability to specifically target [128, 129].

Combining Ad viruses to polyethyleneimine- (PEI-) coated superparamagnetic iron oxide (Fe_3_O_4_) nanoparticles enhances gene transfection efficiency when the vectors are aimed at a specific magnetic field (MGF) located externally [130]. During the past ten years, major advances in oncolytic virus development have allowed for the development of clinical use of OV therapy. Ad-based cancer gene therapy continues to evolve with novel and more cancer cell-specific oncolytic Ads [131]. Choi et al. [132] linked GFP-expressing, replication-incompetent Ad (dAd) with PEGylated and cross-linked iron oxide nanoparticles (PCION), to create dAd-PCION complexes, and found these complexes showing independence of CAR expression and increased transduction efficiency and oncolytic Ad (HmT)-PCION replication inside the cell. The results suggested that MGF-responsive PCION-coated oncolytic Ads might be used as smart complex cancer gene therapy vehicles.

The PEG/lipids/calcium-phosphate- (CaP-) oncolytic adenovirus (PLC-OncoAd) delivery system was constructed for ZD55-IL-24 (an oncolytic adenovirus that carries the IL-24 gene) and was less toxic to the system, lowered liver sequestration, and was not affected by the immune system response. Meanwhile, efficient targeted delivery and improved therapeutic efficacy were achieved without inducing toxicity in hepatocellular carcinoma [133].

This novel transfer system could potentially improve oncolytic adenovirus-based cancer gene therapy. Several studies have described noninvasive imaging of oncolytic viruses [134, 135]. In light of this development, it has become evident that there is a significant need for an exact, responsive, and reproducible way of noninvasively imaging the OV-combined nanoparticles cluster complexes after application to patients.

Although current studies suggest promising future directions, many challenges can arise in actual clinical trials because multiple components exist in these nanostructures, such as species-dependent immune responses, higher toxicities, and the great gaps between the current in vivo mouse model and actual cancer patients imaging which will be very useful or perhaps indispensable in the future cancer detection and management of patients if these current challenges could be overcome.

## Figures and Tables

**Table 1:** Characteristics of molecular imaging modalities and representative examples for nanoparticle-based cancer imaging probes.

Modality	Spatial resolution	Penetration depth	Sensitivity (mol/L)	Cost	Nanomaterial
PET	1-2 mm	No limit	High (10^−11^–10^−12^)	High^*∗∗∗∗*^	Polymer
CT	50–200 *μ*m	No limit	Low (10^−1^–10^−4^)	Low^*∗∗*^	Gold nanoparticle, USPIO nanoparticle
MRI	25–100 *μ*m	No limit	Low (10^−3^–10^−5^)	High^*∗∗∗*^	Paramagnetic liposome, USPIO nanoparticle
US	50–500 *μ*m	mm-cm	Medium	Low^*∗*^	Microbubble

*∗* represents cost value; the more the stars, the higher the price.
